# Behind the seeds: Genetically engineered methionine-rich Arabidopsis seeds show altered metabolism and DNA methylation

**DOI:** 10.1093/plphys/kiad367

**Published:** 2023-06-27

**Authors:** Moona Rahikainen

**Affiliations:** Plant Physiology, American Society of Plant Biologists, Rockville, MD, USA; Organismal and Evolutionary Biology Research Programme, Faculty of Biological and Environmental Sciences, University of Helsinki, Helsinki FI-00014, Finland

Methionine (Met) is an essential amino acid for humans and animals. Plants can synthetize Met, but most tissues have relatively low quantities that limit the nutritional quality of plants and seeds in human diets (Amir 2010). Met has a key role in the translation initiation and structural roles in proteins. In addition, it is essential for many cellular processes as a precursor of S-adenosyl-L-methionine (SAM). SAM is a universal methyl group donor in transmethylation reactions of DNA, RNA, proteins, and metabolites (Roje 2006). Via SAM, Met metabolism is directly linked, for example, to the synthesis of chlorophyll, cell wall polymers, polyamins, and glucosinolates (Roje 2006). Moreover, SAM is a precursor for ethylene synthesis (Sauter et al. 2013). Therefore, Met has a dual role in cells as an essential building block of biomolecules but also as a regulator of cellular functions. Understanding how plants regulate their Met homeostasis is important for breeding more nutritious crops. Equally important is to understand how the changes in cell Met content affect other cellular processes and the productivity and stress tolerance of the whole plant.

In the de novo synthesis of Met, cystathionine-γ-synthase (CGS) catalyzes the synthesis of cystathionine, which is further metabolized to homocysteine and finally to Met (Kim et al. 2002). The key mechanism in the control of cellular Met homeostasis is the SAM-mediated feedback regulation of CGS translation (Onouchi et al. 2004; Hacham et al. 2006). Intriguingly, an additional feedback-insensitive form of CGS has been identified in Arabidopsis (*Arabidopsis thaliana*) (*AtD-CGS*) that allows the generation of transgenic lines accumulating high levels of Met (Hacham et al. 2006; Cohen et al. 2014, 2016). Previously, Cohen et al. (2014) showed that seed-specific expression of *AtD-CGS* under the phaseolin promoter leads to higher content of Met, amino acids, sugars, proteins, and starch in mature Arabidopsis seeds. In this issue of *Plant Physiology*, Girija et al. (2023) describe the underlying mechanisms resulting in the nutrient-rich seed phenotype in these so-called SSE plants.

Girija et al. (2023) show that *AtD-CGS* is highly expressed in seeds in SSE plants, but *AtD-CGS* expression is also elevated in rosette leaves, indicating that the phaseolin promoter is also active in the vegetative tissues of Arabidopsis. Consequently, both seeds and rosette leaves in SSE plants accumulate soluble Met, other amino acids, and soluble metabolites at late stages of development compared with control plants (Girija et al. 2023) (Fig.). Further transcriptomic analysis of the SSE plants revealed that the rosette leaves were adjusting their gene expression to upregulate amino acid synthesis, sugar metabolism, and metabolite transport (Girija et al. 2023). In addition, at late developmental stage, the SSE rosette leaves displayed upregulation of genes associated with senescence and protein degradation, suggesting that the high amino acid content may result in part from degradation of proteins during leaf senescence (Girija et al. 2023). However, despite the activation of senescence-associated genes, no visual symptoms of accelerated senescence in SSE plants were reported (Girija et al. 2023). Taken together, these results show that high Met in leaves of SSE plants is accompanied by vast metabolic rearrangements.

**Figure.**
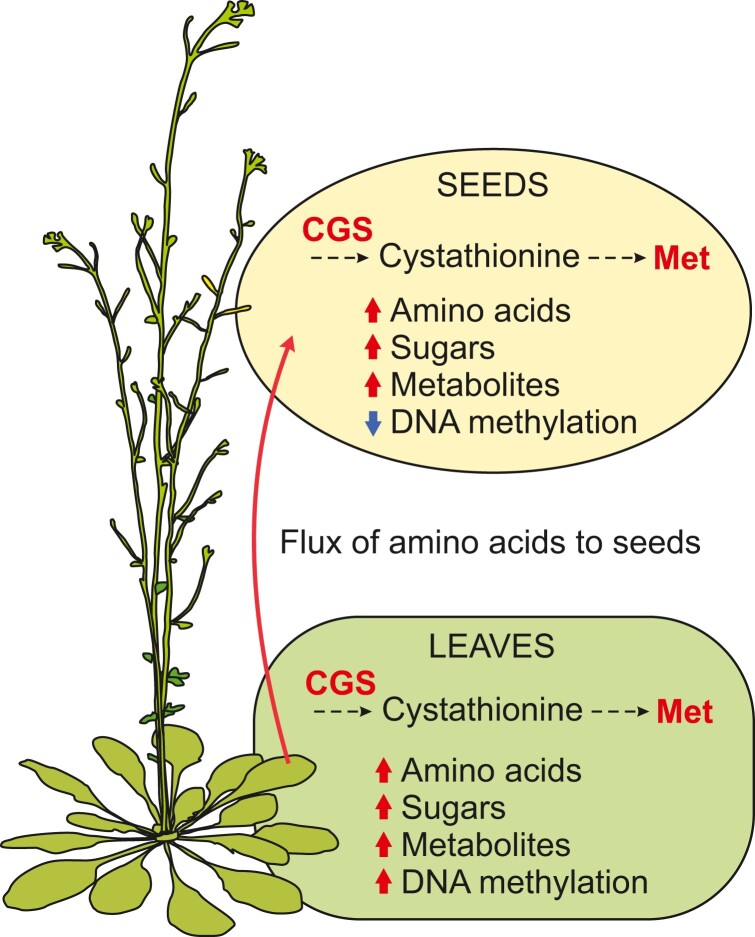
Schematic overview of the physiological changes occurring in the SSE plants. In the de novo synthesis of Met, CGS catalyzes the synthesis of cystathionine, which is further synthesized to homocysteine and finally to Met. In the SSE plants, expression of feedback insensitive *AtD-CGS* results in higher amounts of Met in seeds and rosette leaves. High Met levels promote the accumulation of amino acids, sugars, and other metabolites and increase the flux of amino acids from leaves to seeds. In addition, SSE plants show tissue-specific changes in DNA methylation.

In contrast to leaves, transcriptomic analysis of seeds suggested that the seeds of SSE plants are rather trying to cope with the high levels of Met by upregulating genes associated with Met catabolism and usage (Girija et al. 2023). Notably, the SSE seeds have a low number of upregulated genes associated with amino acid synthesis, suggesting that the observed increase in soluble amino acids was not due to their active synthesis in seeds (Girija et al. 2023). Using isotope labelled [^15^N]Asp or [^15^N]Glu, Girija et al. (2023) were able to show that the flux of amino acids from leaves to seeds is higher in SSE plants compared with control plants. In line with this observation, several genes encoding transporters were activated in leaves and seeds of SSE plants, enhancing the loading of metabolites to phloem and seeds (Girija et al. 2023).

The close connection of Met metabolism to DNA methylation and the high number of differentially expressed genes related to DNA, RNA, and histone methylation in SSE plants prompted Girija et al. (2023) to investigate the changes in DNA methylation in SSE seeds and leaves. Interestingly, the high Met content had distinct effects on DNA methylation in seeds and leaf tissues. At a late stage of development, seeds showed hypomethylation of DNA, whereas SSE leaves tended to have slightly higher DNA methylation compared with control plants (Girija et al. 2023) (Fig.). Alterations in DNA methylation were in accordance with transcriptional changes observed in genes related to methylation reactions that showed downregulation in seeds and upregulation in rosette leaves, respectively (Girija et al. 2023). Taken together, Girija et al. (2023) demonstrate that the metabolic phenotypes of seeds and leaves in SSE plants are associated with tissue-specific alterations in DNA methylation and gene transcription.

This study, together with previous studies on transgenic *AtD-CGS* plants, demonstrates that ectopic expression of the feedback-insensitive CGS is a robust method for Met enrichment of seeds (Cohen et al. 2014, 2016; Girija et al. 2023). In this paper, Girija et al. (2023) show that the Met accumulation causes notable metabolic and transcriptomic rearrangements in both rosette leaves and seeds during late developmental stages. These changes are possibly mediated by organ-specific, Met-induced alterations in DNA methylation and chromatin structural changes (Girija et al. 2023). High Met-induced changes in the activity of methyl transferases and the altered DNA methylation are likely to have indirect effects on many cellular processes. To this end, previous studies have shown that environmental cues affect the germination and metabolic composition of *AtD-CGS* expressing Arabidopsis and soybean (*Glycine max*) seeds, respectively (Cohen et al. 2014, 2016). Thus, future research is needed to investigate how high Met content affects plant development and productivity under fluctuating or stressful environmental conditions.

## Data Availability

No new data were generated or analyzed in this article.
